# A Rare Case of Giant Bilateral Adrenal Myelolipomas in a Patient With Classical Congenital Hyperplasia

**DOI:** 10.7759/cureus.56953

**Published:** 2024-03-26

**Authors:** Meghana Kethireddy, Taejun Lee, Medora Rodrigues, Iqbal Munir, Daniel I Kim

**Affiliations:** 1 Internal Medicine, Rocky Vista University College of Osteopathic Medicine, Saint George, USA; 2 Internal Medicine, Loma Linda University School of Medicine, Loma Linda, USA; 3 Internal Medicine, Loma Linda University Medical Center, Loma Linda, USA; 4 Endocrinology, Riverside University Health System Medical Center, Moreno Valley, USA; 5 Internal Medicine, Riverside University Health System Medical Center, Moreno Valley, USA

**Keywords:** adrenal tumors, adrenal insuficciency, bilateral adrenal masses, adrenal myelolipoma, congenital adrenal hyperplasia ( cah )

## Abstract

Congenital adrenal hyperplasia (CAH) is caused by genetic defects in the enzymes involved in cortisol biosynthesis in the adrenal gland and, in more than 90% of cases, due to a deficiency in the 21-hydroxylase enzyme. Classical CAH due to 21-hydroxylase deficiency is a severe form of the disease that presents with cortisol deficiency and is further categorized into salt-wasting or simple-virilizing types. Appropriate steroid replacement has been shown to effectively treat patients with classical CAH and prevent complications. Individuals who receive inadequate treatment or fail to comply with their prescribed steroid hormone regimen are susceptible to the development of adrenal myelolipomas. Myelolipomas are benign tumors composed of both adipose and hematopoietic tissues. While documented cases of adrenal myelolipomas exist in medical literature, instances of large bilateral myelolipomas remain exceedingly rare.

This case report highlights a 40-year-old female patient with a known history of classical congenital adrenal hyperplasia who presented with unusually large bilateral adrenal myelolipomas. A diagnostic CT scan of the abdomen and pelvis revealed a 13.4 x 10.8 cm myelolipoma on the left adrenal gland and a 10 x 8.6 cm myelolipoma on the right adrenal gland. Prior to her presentation, the patient experienced recurrent nausea and vomiting, along with left upper quadrant pain, over five months. Hormonal assessments indicated significantly elevated serum androgen levels, suggesting inadequate management of her CAH. In this report, we present a rare case of symptomatic bilateral large adrenal myelolipomas, underscoring the significance of adhering to treatment regimens, diagnostic assessments, and management for adrenal myelolipomas in individuals diagnosed with CAH.

## Introduction

Congenital adrenal hyperplasia (CAH) is a rare autosomal recessive disorder affecting the biosynthesis of cortisol [1]. In about 95% of CAH cases, mutations occur in the gene responsible for encoding 21-hydroxylase, causing a loss of enzymatic activity [1,2]. A decreased cortisol level stimulates adrenocorticotropic hormone (ACTH) synthesis. ACTH increases the production of cortisol precursors, which get diverted to synthesize excess androgen [1]. Elevated levels of adrenal androgens can result in abnormal genitalia during female fetal development. Classical CAH represents the more severe form of the disorder and is often subdivided into salt-wasting CAH and simple virilizing CAH, which is a less severe form as patients retain some ability to produce aldosterone. The medical management of classical CAH involves the replacement of synthetic glucocorticoids and mineralocorticoids [1,2]. Non-compliance with the prescribed steroid regimen can lead to adrenal insufficiency or crisis and the exacerbation of adrenal androgen synthesis [1,2].

Elevated circulating ACTH levels commonly seen in patients who are noncompliant with steroid replacement can contribute to adrenal hyperplasia [2]. Approximately 20 to 30% of adult patients with a diagnosis of CAH develop adrenal tumors [3]. Adrenal myelolipomas, which are benign tumors composed of both adipose and hematopoietic tissues, constitute one-fourth of these tumors and can occur unilaterally or bilaterally [1,4]. CAH patients are found to have a markedly elevated risk of developing myelolipomas, exceeding 30 times the risk compared to the general population (10.1% vs. 0.32%) [3]. The majority of adrenal myelolipomas, accounting for approximately 85-90% of cases, are incidentally discovered. They are typically identified during medical imaging procedures conducted for reasons unrelated to adrenal diseases and usually have a median tumor size of 2-2.5 cm during diagnosis [5]. However, when myelolipomas grow larger, exceeding a size of 6 cm, they may become symptomatic [6]. Such symptoms often result from the mass effect exerted by the enlarged tumor and can include abdominal pain, flank pain, or shortness of breath, particularly in certain positions. Additionally, cases of acute hemorrhage requiring surgical intervention have been reported exclusively in patients with large adrenal myelolipomas in approximately 6.8% of cases [6]. Imaging plays a pivotal role in the diagnosis of adrenal myelolipoma. Computed tomography (CT) or magnetic resonance imaging (MRI), with or without contrast enhancement, establishes the diagnosis by identifying macroscopic fat within these tumors [5].

There has been a reported increased incidence of adrenal myelolipomas in patients with excess ACTH production from conditions such as Cushing disease or CAH [5]. The proposed mechanism between endocrine conditions such as CAH and the incidence of myelolipomas is a result of decreased negative feedback to the pituitary gland, resulting in excess ACTH, which stimulates adrenal hyperplasia and possible metaplasia. [5]

This case report was previously presented as a poster at the 2023 American College of Physicians Southern California Chapter Scientific Meeting on October 7th, 2023.

## Case presentation

This case involves a 40-year-old female with a medical history of classical congenital adrenal hyperplasia, reportedly of the salt-wasting type, which was diagnosed shortly after her birth. The exact enzyme deficiency responsible for her condition remains unknown. However, her medical history is most consistent with a severe 21-hydroxylase deficiency. She was born with ambiguous genitalia and underwent corrective genital surgery shortly after birth. Unfortunately, medical records dating back to her birth were unavailable, and the earliest record accessible for review was from when she was 15 years old, during a urology follow-up appointment.

According to the available medical record, the patient had undergone two genital surgeries: the first at around 18 months old, which involved a feminizing genitoplasty, and the second when she was approximately seven years old, aimed at removing a cystic structure that was causing pseudo-enlargement of the clitoris. Notably, during this period, she was taking hydrocortisone and fludrocortisone. Additionally, the patient has a medical history of attention deficit hyperactivity disorder (ADHD), schizophrenia, and a substance use disorder involving alcohol and methamphetamines.

Her initial presentation to the hospital was due to symptoms of recurrent nausea, vomiting, and left upper quadrant pain. These symptoms started five months earlier but had worsened significantly over the past two weeks, prompting her to seek medical attention. She reported an increased consumption of calcium carbonate, bismuth subsalicylate, and ibuprofen during this time in an attempt to alleviate the pain and vomiting, albeit with minimal relief. The patient noted that both eating and drinking exacerbated her symptoms. She confirmed her current prescriptions, which included hydrocortisone at a dosage of 5 mg three times daily and fludrocortisone at 100 mcg once daily. Due to the patient’s history of ADHD, she also endorsed having a prescription and taking extended-release amphetamine-dextroamphetamine at 20 mg twice daily. However, she admitted to non-adherence with her hydrocortisone and fludrocortisone medications over the last three months due to increased nausea and vomiting.

On a physical exam, the patient’s abdomen was mildly tender to palpation in the left upper quadrant. Her abdomen was non-distended and soft, and bowel sounds were present in all four quadrants. In addition, her buccal mucosa and skin showed no evidence of hyperpigmentation. On presentation, her vitals were reported as the following: blood pressure of 130/70, heart rate of 115 bpm, respiratory rate of 16, oxygen saturation of 99% on room air, and a temperature of 97.8 F. Lab studies during the initial presentation revealed electrolyte abnormalities including mild hyponatremia, hypokalemia, hypomagnesemia, hypophosphatemia, a mild elevation in serum creatinine, and a normal bicarbonate level, as seen in Table 1. She had severely elevated serum calcium at presentation, likely from dehydration and consumption of excessive amounts of calcium, which resolved after initial fluid resuscitation. Further evaluation showed endocrine function abnormalities consistent with CAH, as demonstrated in Table 2. 

**Table 1:** Metabolic panel lab values on initial presentation reflecting several electrolyte abnormalities. Na^+^: sodium; ​​​​​​​K^+^: potassium; Cl ^-^: chloride; Bicarb: bicarbonate; Cr: creatinine; Ca: calcium; Phos: Phosphorous; Mg: magnesium

Electrolytes							
Na^+^	K^+^	Cl^-^	Bicarb	Cr	Ca	Phos	Mg
(normal 135-145 mEq/L)	(normal 3.4 -5.0 mmol/L)	(normal 96-106 mEq/L)	(normal 22 – 32 mmol/L)	(normal 0.6 – 1.1 mg/dL)	(normal 8.6 - 10.3 mg/dL)	(normal 2.8 -4.5 mg/dL)	(normal 1.7 – 2.2 mg/dL)
131 mEq/L	2.4 mmol/L	93 mEq/L	26 mmol/L	1.2 mg/dL	13.8 mg/dL	2.1 mg/dL	1.3 mg/dL

**Table 2:** Endocrine studies drawn one day after admission. ACTH: adrenocorticotropic hormone; DHEA-S: dehydroepiandrosterone sulfate; 17-OHP: 17-hydroxyprogesterone

Endocrine Studies					
ACTH	DHEA-S	17-OHP	Androstenedione	Testosterone	Renin
(normal 6 – 50 pg/dL)	(mid-follicular: 385- 1143 ng/dL) (post-menopausal: 77-851 ng/dL)	(normal < 200 ng/dL)	(normal 6 – 86 ng/dL)	(normal 2-45 ng/dL)	(normal 0.25 – 5.82 ng/dL)
18 pg/dL	<20 ng/dL	927 ng/dL	214 ng/dL	142 ng/dL	1.24 ng/dL

In the emergency department, her symptoms started to improve following IV fluid administration and electrolyte replacement; a stress dose of steroid was not administered. She was started on a regimen of hydrocortisone at 5 mg three times a day (TID). Once her hypokalemia resolved, she was started on a daily dose of fludrocortisone at 100 mcg.

**Figure 1:**
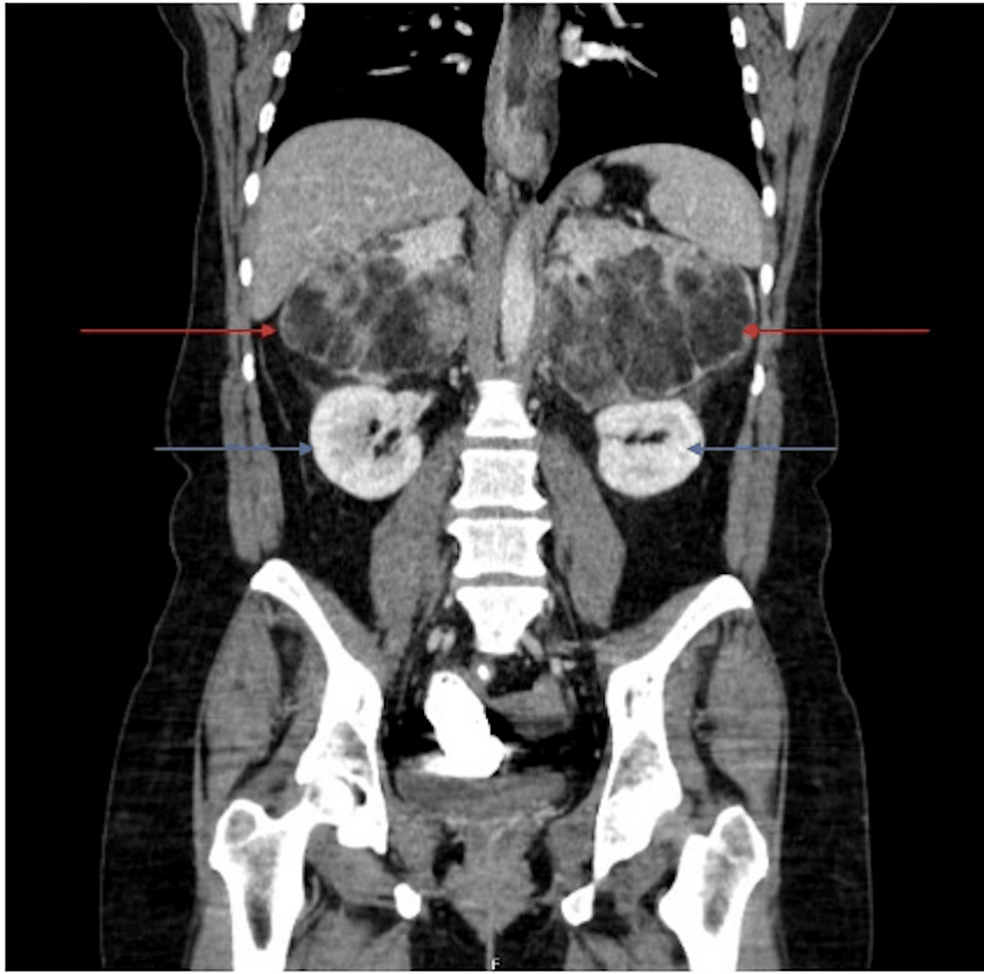
Anterior view of CT of abdomen and pelvis with contrast. Bilateral adrenal myelolipomas (red arrows) and inferiorly deviated kidneys are visible (blue arrows). CT: computed tomography

**Figure 2:**
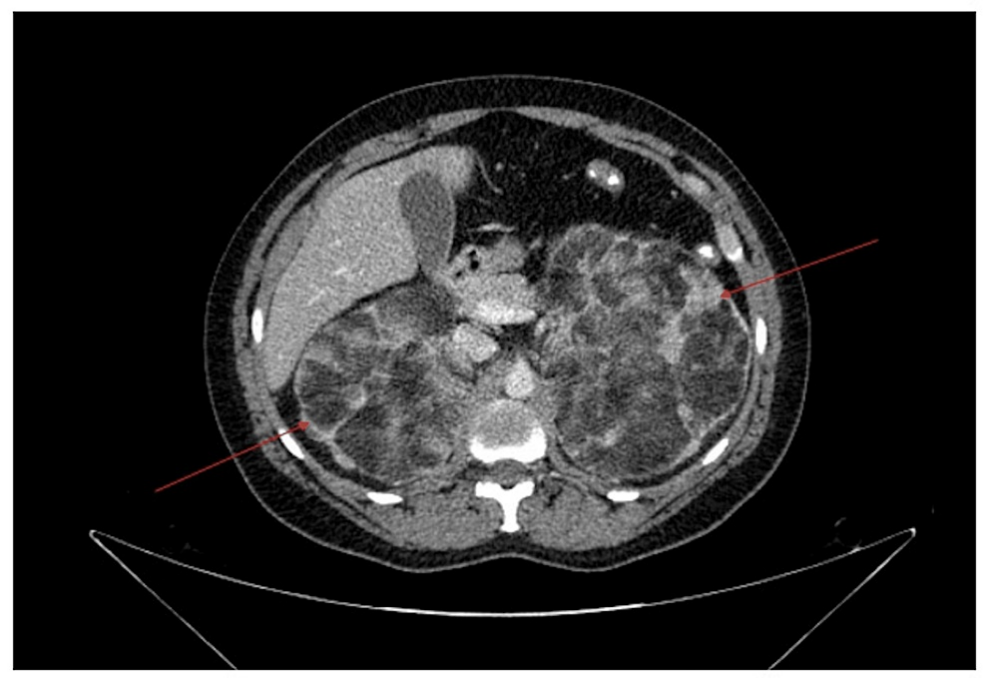
Cross section of CT abdomen and pelvis with contrast. Bilateral fat-containing adrenal masses characteristic of adrenal myelolipomas (red arrows) are visible. CT: computed tomography

Imaging of the abdomen included a CT scan of the abdomen and pelvis with contrast (Figure 1, 2). A CT scan showed bilateral fat-containing adrenal lesions characteristic of adrenal myelolipoma, with the left measuring 13.4 x 10.8 cm and the right measuring 10 x 8.6 cm. The myelolipomas had a mass effect on the kidneys, which were deviated inferiorly.

## Discussion

We presented a case with large bilateral adrenal myelolipomas in a patient with a documented history of classical congenital adrenal hyperplasia (CAH) and a pattern of inconsistent medication adherence. Her endocrine studies completed in the early morning, the day after the initial presentation, revealed elevated levels of androstenedione, 17-hydroxyprogesterone, and total testosterone. These findings are consistent with the patient's history of irregular use of a steroid regimen to manage her CAH. It's important to note that increased levels of androgen precursors, such as androstenedione and 17-hydroxyprogesterone, can be converted into testosterone, thus explaining the increased testosterone levels observed in this patient. Notably, her dehydroepiandrosterone sulfate (DHEA-S) level remained within the normal range, a phenomenon often observed in CAH patients [7].

Interestingly, the patient's serum adrenocorticotropic hormone (ACTH) level obtained in the morning after admission did not show elevation during this hospital admission, possibly attributed to taking hydrocortisone or for unclear reasons. Additionally, her plasma renin activity appeared normal, suggesting a restoration of adequate volume status.

This patient's history is marked by a longstanding pattern of noncompliance with her prescribed steroid regimen for CAH. She reported adhering to her medications consistently during her youth but admitted to taking them irregularly during adulthood, including stopping medications periodically for up to several months. A review of her medical records reveals endocrine laboratory results from when she was 24 years old, showing evidence of poorly controlled hormone levels, including an exceptionally elevated ACTH level of 801 pg/mL, androstenedione at 1829 ng/dL, 17-hydroxyprogesterone at 30,111 ng/dL, and a total testosterone level of 411 ng/dL.

Interestingly, the patient has not experienced an adrenal crisis during her adult years, despite her history of noncompliance with medication. This admission also did not involve an adrenal crisis. It's worth noting that many adult patients with CAH who do not adhere to glucocorticoid treatment can go for extended periods without experiencing adrenal crises [8]. This phenomenon can be puzzling to comprehend.

The reasons behind why some CAH patients can go for years without requiring glucocorticoids remain unclear. In vitro studies have demonstrated that certain adrenal steroid precursors, including 17-hydroxyprogesterone, which accumulate in high quantities in classic CAH, can act as partial glucocorticoid agonists [9]. Additionally, some of these adrenal steroid precursors that accumulate in CAH can interact with glucocorticoid and mineralocorticoid receptors as antagonists and agonists [9,10].

There is an association between chronic ACTH stimulation and a higher risk of developing adrenal myelolipomas, as it is more common in conditions of excess ACTH like CAH and Cushing disease [5, 6]. Not all patients with myelolipoma have excess ACTH levels [5]. Metaplasia of the reticuloendothelial cells of the blood capillaries in the adrenal gland is a widely accepted theory in the pathogenesis of myelolipoma [5, 11]. Medication nonadherence by our patient likely resulted in long periods of ACTH overstimulation and, consequently, the development of large bilateral adrenal myelolipomas.

Though the CT scan is the most sensitive test in diagnostic evaluation for adrenal myelolipoma, it is important to address differential diagnoses that may present similarly on computed tomography [4]. Other potential diagnoses include adrenal angiomyolipoma, liposarcoma, and adenoma. The key characteristic in differentiating these pathologies from a myelolipoma is the presence of mature adipose tissue, which results in lower densities in the tumor in a CT scan. Though adrenal adenomas also present with lower densities, they present as homogenous structures compared to myelolipomas, which are heterogenous [4]. In addition, myelolipomas present with well-defined capsules of soft tissue as opposed to a liposarcoma that would present with an infiltrative pattern in the CT scan [12]. Angiomyolipomas on the adrenal gland are very rare and are more commonly seen in the kidney. Further coronal CT reconstruction or MRI could be used to determine if the mass originated in the kidney. Another diagnostic tool to differentiate the pathologies is a tissue biopsy. Due to the large number of myeloid cells and the location of the tumor, biopsy is not recommended for the diagnosis of adrenal myelolipomas because it carries a large risk of retroperitoneal hemorrhage. However, fine needle aspirations are recommended if there is suspicion of malignant etiologies of the mass [12]. 

Management for myelolipomas varies based on the symptoms and sizes of the masses. For asymptomatic myelolipomas under 4 cm, the recommendations are conservative, whereas symptomatic tumors or myelolipomas over 7 cm are recommended for surgical excision to prevent the risk of spontaneous rupture causing retroperitoneal hemorrhage [12]. The risks and benefits of an adrenalectomy must be taken into account. Current studies support a laparoscopic approach to adrenalectomy due to the reduced operating time, decrease in blood loss, and similar success rate compared to open procedures [13]. Hormone replacement for patients who undergo adrenalectomy is imperative to prevent complications such as adrenal insufficiency and crisis. The decision for surgical excision is a shared decision between the provider and the patient. In this case, a consultation with surgical oncology was placed, and a bilateral adrenalectomy was recommended.

Adrenalectomy can also be valuable in ameliorating virilization resulting from excessive adrenal androgen production as well as iatrogenic Cushing syndrome, which may develop as a consequence of administering supraphysiological steroid treatments to suppress androgen production in these patients [7]. Certainly, adrenalectomy could be instrumental in not only improving abdominal symptoms caused by the large myelolipoma but also in resolving hyperandrogenemia in our patient.

## Conclusions

This case involves a 40-year-old woman who has a medical history of classical congenital adrenal hyperplasia (CAH), most likely caused by a deficiency in the 21-hydroxylase enzyme and a history of non-compliance with her steroid regimen. She presented with very large bilateral adrenal myelolipomas. Giant adrenal myelolipomas are uncommon tumors composed of a mixture of hematopoietic and adipose tissue. The presence of adrenal myelolipomas in a patient with CAH is in line with the suggested mechanism of chronic stimulation by adrenocorticotropic hormone (ACTH). Due to the substantial size of the tumors in this patient, it was recommended that surgical removal of the adrenal glands be done.
